# Anti-tumor effects of PEGylated-nanoliposomes containing ginger extract in colorectal cancer-bearing mice

**DOI:** 10.22038/IJBMS.2022.63870.14075

**Published:** 2022-07

**Authors:** Maryam Yavari, Mahmoud Reza Jaafari, Farshad Mirzavi, Ghasem Mosayebi, Ali Ghazavi, Ali Ganji

**Affiliations:** 1 Department of Immunology & Microbiology, School of Medicine, Arak University of Medical Sciences, Arak, Iran; 2 Nanotechnology Research Center, Mashhad University of Medical Sciences, Mashhad, Iran; 3 Department of Clinical Biochemistry, Faculty of Medicine, Mashhad University of Medical Sciences, Mashhad, Iran; 4 Cardiovascular Diseases Research Center, Birjand University of Medical Sciences, Birjand, Iran; 5 Molecular and Medicine Research Center, Arak University of Medical Sciences, Arak, Iran; 6 Traditional and Complementary Medicine Research Center (TCMRC), Arak University of Medical Sciences, Arak, Iran

**Keywords:** Bax protein, Colorectal cancer, Ginger, Interferon-gamma, Liposomes

## Abstract

**Objective(s):**

This study aimed to develop a nanoliposomal formulation containing ginger ethanolic extract with a higher therapeutic effect for cancer treatment.

**Materials and Methods:**

The present study aimed to prepare PEGylated nanoliposomal ginger through the thin film hydration method plus extrusion. Physicochemical characteristics were evaluated, and the toxicity of the prepared liposomes was assessed using the MTT assay. In addition, tumor size was monitored in colorectal cancer-bearing mice. Also, the anticancer effects of liposomal ginger were evaluated by gene expression assay of Bax and Bcl-2 and cytokines including TNF-α, TGF-β, and IFN-γ by Real-time PCR. Also, cytotoxic T lymphocytes (CTLs) and regulatory T lymphocytes (Treg cells) were counted in spleen and tumor tissue by flow cytometry assay.

**Results:**

The nanoliposomes’ particle size and polydispersity index (PDI) were 94.95 nm and 0.246 nm, respectively. High encapsulation capacity (80 %) confirmed the technique’s efficiency, and the release rate of the extract was 85% at pH 6.5. In addition, this study showed that liposomal ginger at 100 mg/kg/day enhanced the expression of Bax (*P<*0.05) and IFN-γ (*P<*0.01) compared with ginger extract in the mouse model. Also, the number of tumor-infiltrating lymphocytes (TILs) and CTLs cell count in tumor tissue showed a significant increase in the LipGin group compared with the Gin group (*P<*0.05).

**Conclusion:**

Results indicated that the liposomal ginger enhanced the antitumor activity; therefore, the prepared liposomal ginger can be used in future clinical trials.

## Introduction

Colon cancer is the third leading cause of cancer mortality globally and steadily increases in developing countries (1, 2). Currently, chemotherapy, radiation therapy, and surgery are used for colorectal cancer treatment. However, due to chemotherapy’s adverse side effects on normal cells and acquired resistance to the therapeutic agents, the tendency to use herbal medicine in addition to conventional therapies has been increased (3, 4). Hence, researchers use herbal compounds to reduce unwanted side effects and improve the treatment’s effectiveness (5-7). 

Ginger or* Zingiber officinal* with active ingredients including gingerol and shagoal has several activities, including anti-inflammatory, antioxidant, and anticancer effects. Previous research has shown evidence for the preventive and therapeutic effects of ginger extract and its active ingredients using *in vitro* and *in vivo* studies (8). Ginger plays anticancer effects via induction of cancer cell death, cell cycle arrest, metastasis inhibition, and angiogenesis prevention (9-11). Ginger has antitumor activity against gastrointestinal cancer by modulating signaling molecules, inflammatory cytokines, caspase molecules, and proteins involved in cell growth regulation (8). Although ginger’s favorable effects have been observed, its use has been challenged due to its early degradation and non-targeted delivery to the tumor site. Using nanoparticles, especially liposomes, can overcome many problems that lead to a practical therapeutic approach (12, 13).

Liposomes are spherical vesicles made of lipids similar to the cell membrane with low toxicity and ease of production methods (14, 15). Their amphipathic properties can encapsulate hydrophilic drugs in the aqueous center and hydrophobic drugs between lipid bilayers. It is also possible to use a polymer compound such as polyethylene glycol (PEG) to coat the liposomes, which causes the liposomes to escape from reticuloendothelial system (RES) clearance and increases the half-life of the circulating liposomes (16, 17). Liposomes with long circulation times leak preferentially into tumor tissue through the permeable tumor vasculature and are then retained in the tumor microenvironment due to reduced lymphatic drainage. This phenomenon is known as the enhanced permeability and retention (EPR) effect (18, 19). Therefore, using these nanoparticles as a drug delivery system can cause targeted delivery to the tumor, protect drug compounds from premature destruction, and increase drug bioavailability (20).

Although many studies prove ginger’s anticancer effect, no study has yet evaluated the antitumor effects of liposomal ginger. Therefore, this study aimed to prepare PEGylated-liposomes containing alcoholic ginger extract to evaluate the anticancer and immune system stimulatory effects in cell culture and mouse models of colon cancer.

## Materials and Methods

***Ginger extract preparation***

Ginger’s dried rhizomes were purchased from a reputable store and approved by the herbarium of Arak University of Medical Sciences. Dried ginger rhizome (25 gr) was ground and extracted with 250 ml of absolute ethanol (1:10) in the Soxhlet apparatus (Laborota 4000, Heidolph, Germany) for 4 hr at 78.9 ^°^C (9). Then, the ethanol in the extract was evaporated by a rotary evaporator (Laborota 4000, Heidolph, Germany) at 89 ^°^C. Finally, the obtained extract was dissolved in dimethyl sulfoxide (DMSO) and kept dark at -20 ^°^C until use.

***Liposome preparation and characterization***

Liposomes containing ginger (LipGin) were prepared by the hydration method. In this way, hydrogenated soybean phosphatidylcholine (HSPC) (Ludwigshafen, Germany), cholesterol (Sigma-Aldrich, Mo), and mPEG2000-DSPE (Ludwigshafen, Germany) were dissolved in chloroform with 50 mM total lipid concentration [HSPC/chol/mPEG2000-DSPE (55:30:5 mol/mol)]. Then, ethanolic ginger extract (Gin) (10% w/w) was added to the lipid phase. The lipid dissolution in chloroform was removed under a rotary evaporator, followed by freeze-drying. In the next step, the lipid film was hydrated with a stabilizing buffer containing sucrose (280 mM) and histidine (10 mM) at pH=6.5 (21). Then the hydrated liposome was sonicated at 65 ^°^C for 40 min in a bath sonicator (Bandelin, Germany). Next, the formulations were extruded under argon gas pressure to decrease the size of liposomes by 400, 200, and 100 nm polycarbonate membranes. Finally, the unencapsulated ginger extract was removed by dialysis against a hydration buffer at 4 ^°^C by a 100 kDa dialysis bag (Spectra/Por™ 2 12-14 kD MWCO). A dynamic light scattering device measured particle size, polydispersity index (PDI), and the liposomes’ zeta potential (DLS, Nano-ZS; Malvern, UK). According to the approved protocol, the morphology of the liposomes containing ginger extract was assessed by negative staining using atomic force microscopy (AFM) (Zeiss, Jena, Germany). 

***Extract encapsulation efficiency***

Encapsulation efficiency (EE) was reported as the ratio of encapsulated extract to the total extract in percentage. Hence, the prepared suspension containing liposomal ginger was first centrifuged at 14000 rpm for 10 min to separate the unencapsulated ginger (sample 1). Then, to lyse the liposomes and release the ginger, methanol was added to the residue at a dilution of 1 to 50 and sonicated for 5 min. Then the lysed sample was centrifuged at 14000 rpm for 10 min (sample 2). Finally, the absorption of ginger in samples 1 and 2 was read at a wavelength of 226 nm with a UV-visible spectrophotometer (SPEKOL 1300; Analytik Jena, Germany) compared with the extract’s standard curve. The encapsulation percentage was calculated using the following formula: EE %= encapsulated extract (mg) / amount of primary extract (mg) × 100.

***Extract release rate***

For this purpose, 0.5 ml of the prepared formulation was poured into three dialysis bags with 3.5 kD molecular weight cut off (Pierce, Rockford, IL) and placed in buffers with different pH, including PBS (pH=7.5), sucrose/histidine (pH=6.5) and sucrose/histidine (pH=5.5). Extraction release was measured at other times (0.5, 1, 2, 4, 8, 12, and 24 hr) through optical absorption of all samples at 226 nm by a spectrophotometer.

***MTT assay***

For the MTT assay, C26 cells were obtained from the Pasture Institute (Tehran, Iran). The cells were cultured in a complete RPMI-1640 medium (Gibco, USA) enriched with 10% FBS (Gibco, USA), 100 IU/ml penicillin, and 100 mg/ml streptomycin (Gibco, USA). The cells were seeded in a 96-well microplate (SPL Life Sciences, Korea) and incubated overnight at 37 ^°^C and 5% CO_2_. Then, the cultured cells were treated with 50–800 μg/ml of LipGin and Gin for 48 hr in triplicate. After incubation, 5 mg/ml of MTT (Sigma, USA) was added to each well, and the cells were incubated for 4 hr. Then, 200 μl of DMSO (Sigma, USA) was added to each well. After shaking for 45 min in the dark, the formed purple color of formazan crystals in living cells was measured at 560 nm, and IC_50_ was calculated in Excel software based on the linear equation. Finally, cell viability was calculated using the following formula: Cell Viability%=OD treated cells/ OD control×100 (9). Also, mouse splenocytes were selected as normal cells and collected according to the previous protocol (22). Splenocytes, like C26 cells, were treated to similar concentrations of LipGin and Gin for 48 hr in triplicate, and the MTT assay was performed as mentioned above.

***Mouse model***

In this study, 4-6 week-old female Balb/c mice were purchased from the Pasteur Institute (Tehran, Iran) and kept in a 12/12-hr light/dark cycle at 22-25 ^°^C with free access to water and food. Mice were maintained and examined based on the Animal Ethics Committee of Arak University of Medical Sciences protocol (IR.ARAKMU.REC.1398.336). Colorectal cancer is induced subcutaneously (SC) in mice by injecting 3×10^5 ^C26 cells into the body’s right flank. Treatment began in groups one week after tumor induction when the tumors became palpable. Mice-bearing tumors were treated for two weeks with four intravenous injections (twice a week). First, the liposomal ginger (LipGin) group was injected with 100 mg/kg/day of liposomes encapsulated ginger. Second, the ginger extract (Gin) group was injected with 100 mg/kg/day of ginger extract. Third, the Doxil group was injected with 2.5 mg/kg/day of Doxil. Fourth, Doxorubicin (Dox) group was injected with 2.5 mg/kg/day of doxorubicin (23), and the control group was injected with PBS. During the 14-day treatment period, mice’s tumor size and weight were measured using calipers and scales. Finally, tumor tissues were removed, weighed, and sized using the following equation: Tumor volume=length×(width×2)×0.52.

***Toxicity evaluation of the injected liposomes***

For this purpose, on the 14^th^ day of treatment, the mice were anesthetized and blood samples collected for estimation of both liver and renal functions by determining the levels of blood urea nitrogen (BUN), creatinine, serum glutamic oxaloacetic transaminase (SGOT), and serum glutamic pyruvic transaminase (SGPT) (Pars Azmoon, Iran).

***Hematoxylin and eosin (H&E) staining***

Tumor tissues isolated from mice were first fixed in formalin and molded into paraffin blocks for incision. Next, tumor-infiltrating lymphocytes (TILs) were determined in the tumor tissue by H&E staining.

***Flow cytometry***

Removed tumor tissue and spleen were washed with sterile PBS, and single cells were harvested using a 70 µm cell strainer (SPL Life Sciences, South Korea). For immunophenotyping of cytotoxic CD8 T lymphocytes (CTL), FITC-conjugated mouse anti-CD3 antibody and PerCP-conjugated mouse anti-CD8 antibody were used. Also, PE-conjugated murine anti-CD4 antibody, PerCP-conjugated murine anti-CD25 antibody, and FITC-conjugated murine anti-FoxP3 antibody were used to evaluate T regulatory lymphocytes (Treg cells). All of the compounds were obtained from eBioscience (USA), and flow cytometry was performed by counting 10,000 events based on the manufacturer’s instructions. Finally, the output data were analyzed by Flowjo software (Tree Star Inc., OR, USA).

***Real-time PCR assay***

Gene expression of Bax and Bcl-2 in tumor tissue and TNF-α, TGF-β, and IFN-γ in splenocytes were evaluated using real-time PCR. For this purpose, RNA extraction and cDNA synthesis were performed according to the manufacturer’s protocol (Yekta Tajhiz, Iran). Forward and reverse primers of target and reference (Gapdh) genes were designed using AlleleID 6.0 (Premier Biosoft International, USA), and the sequence of the primers was checked by NCBI Blast for specificity (Table 1). The real-time PCR assay was run in duplicate using SYBR Green I 2x Master Mix (Yekta Tajhiz, Iran) in a LightCycler 96 system (Roche, Switzerland). PCR products were evaluated by the melting curve to confirm the absence of nonspecific products. Quantitative determination of target genes was also performed using the Pfaffle method. 

***Statistical analysis***

Statistical analyses were performed using GraphPad Prism 6.0 software (San Diego, CA, USA). The assumption of normality was tested using the Kolmogorov-Smirnov test. One-way ANOVA and Tukey’s tests were also performed to evaluate differences in variables between the studied groups. Data are presented as Mean±SEM. A *P*-value less than 0.05 was considered statistically significant.

## Results

***Liposomes characterization***

**Figure 1:**
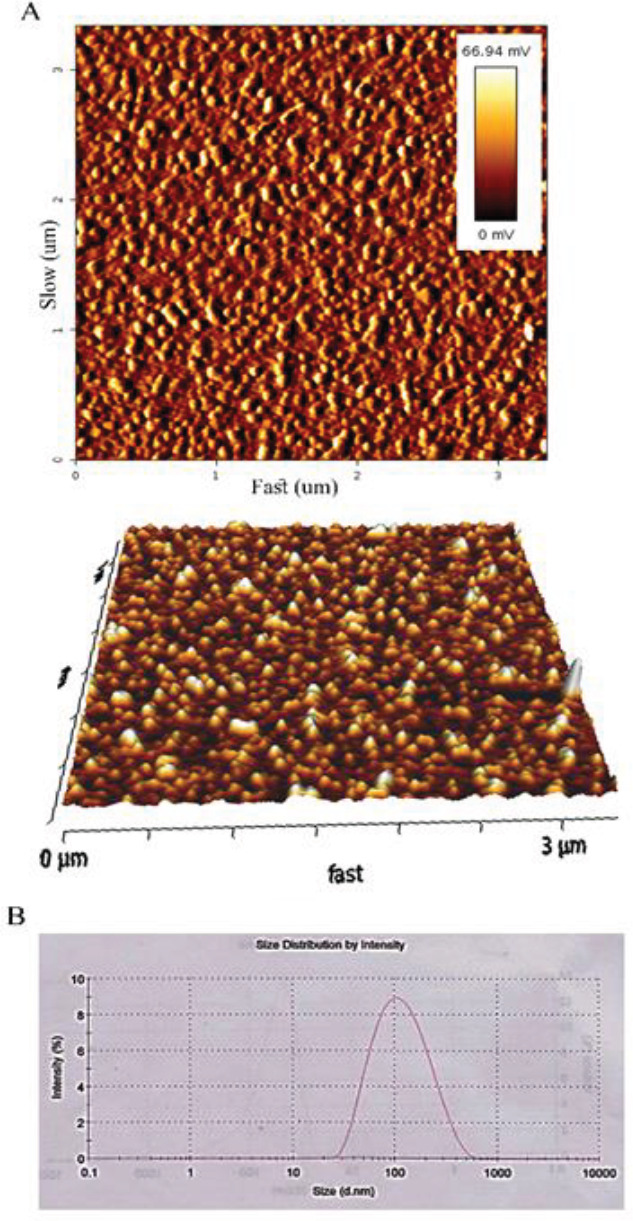
AFM images of ginger-loaded nanoliposomes

**Figure 2:**
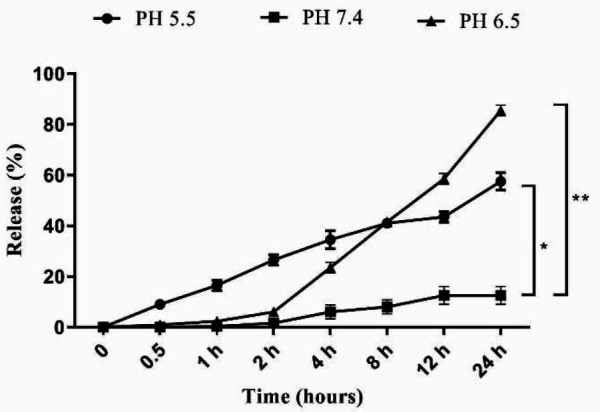
*In vitro* release profile of ginger from liposomal formulation at pH 5.5, 6.5, and 7.4 during 24 hr. Values are presented as mean±standard error of mean vs control group. (*P<0.05) and (**P<0.01)

The atomic force microscopy (AFM) image showed that liposome sizes were around 100 nm (Figure 1A). The particle size diagram obtained from the zeta sizer was 94.95 nm, PDI was 0.246, and the average zeta potential of particles was -11.1 (Figure 1B). Also, the encapsulation efficiency was 80 %. The release profiles of the formulation in 24 hr are shown in Table 2. The release of ginger from LipGin nanoliposomes at pH =6.5 (85.5%) and pH=5.5 (57.5%) was significantly (*P*<0.05) faster than the release of the extract at pH= 7.4 (12.5%) after 24 hr. (Figure 2).

***Cytotoxicity effect of the nanoliposomes***

**Figure 3:**
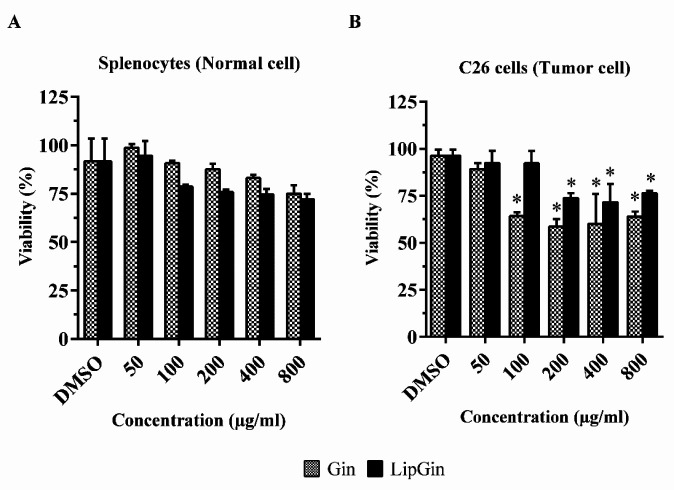
Cytotoxicity effect of liposomal ginger and ginger extract after 48 hr on C26 cell line. Values are presented as mean±standard error of mean vs control group. (*P<0.05) and (**P<0.01)

Statistical results show that the viability of splenocytes as normal cells treated with LipGin and Gin is not significantly different from the control group (Figure 3A). Also, cancer cells’ survival showed a significant reduction in cells treated with 100 μg/μl of Gin and 200 µg/μl of LipGin to the end concentration than the control group (*P*<0.05) (Figure 3B). IC_50_ values for LipGin and Gin groups were 240 and 170 μg/ml, respectively.

***Side effects of the prepared nanoliposomes***

**Figure 4:**
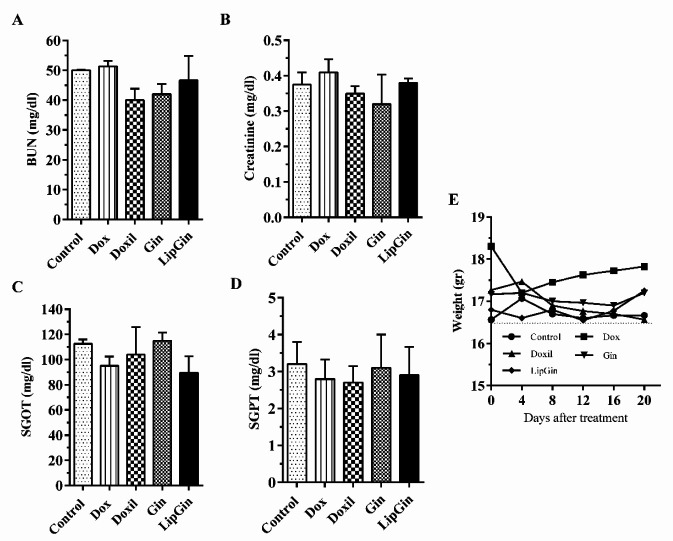
Effect of the extract of ginger on the liver and kidney via (A) BUN (Blood urea nitrogen), (B) Creatinine, (C) SGOT (Serum glutamic oxaloacetic transaminase), and (D) SGPT (Serum glutamic pyruvic transaminase) measurement. (E) Effects of liposomal ginger and ginger extract on body weight in C26 tumor-bearing mice. Values are presented as mean ± standard error of mean vs control group

As shown in Figure 4, no significant difference was observed in the treated groups than the control in BUN, creatinine, SGOT, and SGPT levels (Figures 4A-D) (*P*>0.05). Also, mice’s weight in different groups showed insignificant changes compared with the control (Figure 4E) (*P*>0.05). 

***Evaluation of tumor growth inhibition***

**Figure 5:**
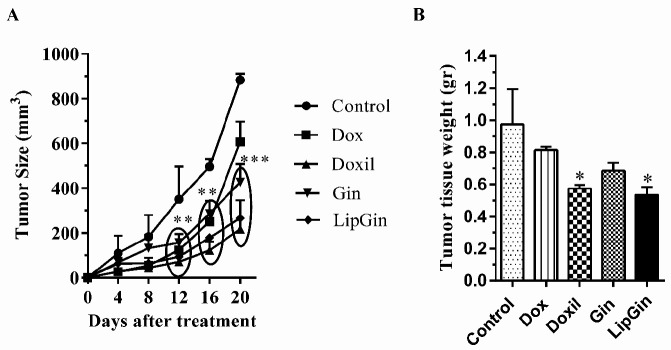
Effects of liposomal ginger and ginger extract on tumor growth. (A) Tumor size monitoring in all groups. (B) Tumor tissue weight in C26 tumor-bearing mice. Values are presented as mean±standard error of mean vs control group. (*P<0.05), (**P<0.01), and (***P<0.001)

Tumor size in Gin and LipGin groups from the 12^th^ day of treatment until the end was significantly reduced compared with the control group (Figure 5A) (*P*<0.05). In addition, tumor tissue weight was decreased considerably in Doxil and LipGin groups than in the control group (*P*<0.05). Tumor weight in the Gin group decreased more than in the control; however, this was not statistically significant (Figure 5B).

***Infiltrated lymphocytes into tumor tissue***

**Figure 6:**
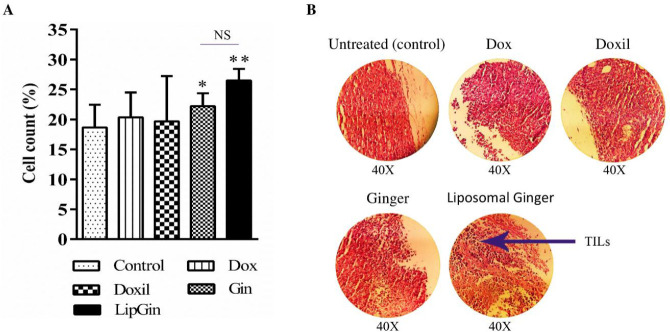
H&E staining of tumor infiltrated lymphocyte. (A) Histogram graph of the different treated groups. (B) H&E staining of histological sections taken from tumors of mice. Values are presented as mean±standard error of mean vs control group. (*P<0.05) and (**P<0.01)

H&E staining of the tumor tissue sections showed a significant increase in the number of tumor-infiltrating lymphocytes (TILs) toward the tumor site in Gin and LipGin groups (*P*<0.05) (Figure 6A). However, there is no significant difference between the other groups and the control (*P*>0.05) (Figure 6B).

***CTLs and Treg cells count in tumor tissue and spleen***

**Figure 7:**
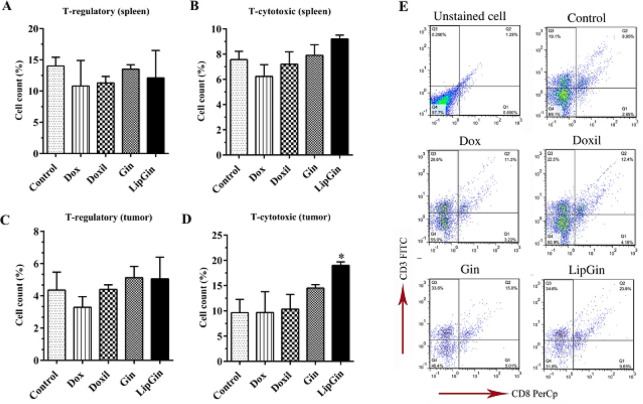
Assessment of the T cell subtype proportions in spleen and tumor tissue. (A) Treg cells and (B) CTLs in mouse spleen. (C) Treg cells and (D) CTLs in tumor tissue. (E) Gating strategies for practical flow cytometry data analysis for CTL cell populations in tumor tissue

Flow cytometry data showed a significant increase in the number of CTLs in the LipGin group compared with the control in tumor tissue (*P*<0.05). There was no significant difference between the other groups and the control (*P*>0.05) (Figures 7A-E).

***Bax and Bcl-2 genes expression***

**Figure 8:**
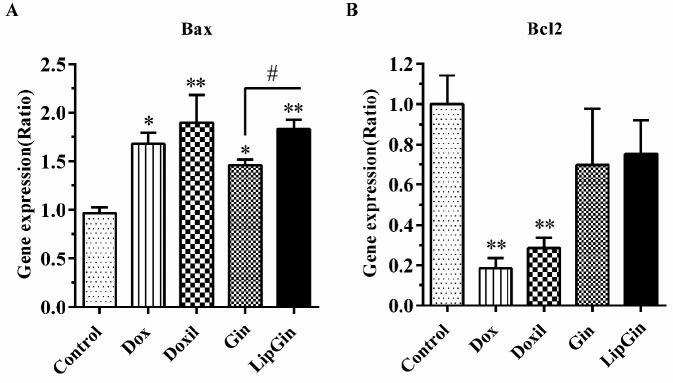
Effects of liposomal ginger on the expression of apoptosis-related genes. Gene expression results of (A) Bax and (B) Bcl-2 genes in tumor tissue. Values are presented as mean±standard error of mean vs control group. (*P<0.05) and (**P<0.01)

The results of real-time PCR showed that Bax gene expression was significantly increased in all groups compared with the control (*P*<0.05). Also, there was a significant increase of the Bax gene in the LipGin group compared with the Gin group (*P*<0.05) (Figure 8A). On the other hand, there was a significant decrease in the expression of the Bcl-2 gene in the Dox and Doxil groups (*P*<0.05), without any significant difference between LipGin and Gin compared with the control (*P*>0.05) (Figure 8B).

***TNF-α, TGF-β, and IFN-γ gene expression***

**Figure 9:**
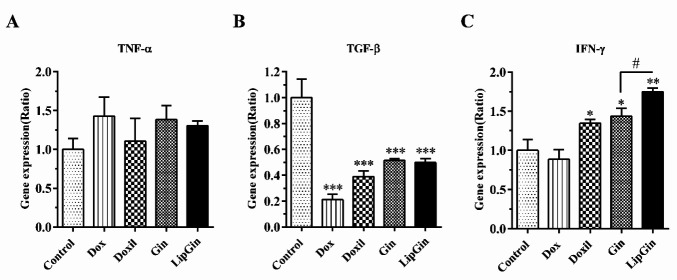
Effects of liposomal ginger on the gene expression of TNF-α, TGF-β, and INF-γ. (A) TNF-α gene expression, (B) TGF-β gene expression, and (C) INF-γ gene expression in splenocytes. Values are presented as mean±standard error of mean vs control group. (*P<0.05), (**P<0.01), and (***P<0.001). * significant results compared with the control. # significant results of LipGin group compared with Gin group

Gene expression of the cytokines in splenocytes showed no significant difference in TNF-α between the groups (Figure 9A) (*P*>0.05). There was a significant decrease in TGF-β gene expression in all groups compared with the control (Figure 9B) (*P*<0.05). There was a significant increase in IFN-γ gene expression in Doxil, Gin, and LipGin groups. There was also a considerable increase in the LipGin group compared with the Gin group (Figure 9C) (*P*<0.05). 

**Table 1:** PCR primers sequences used for gene expression analysis

**Gene**	**Length**	**Primers**	**Sequence, 5′→3′**
**Bax**	174	Forward Reverse	GCTACAGGGTTTCATCCAG TCCACGTCAGCAATCATCC
**Bcl-2**	161	Forward Reverse	TGTGGCCTTCTTTGAGTTCG GTTCCACAAAGGCATCCCAG
**TNF-α**	201	Forward Reverse	CCTCTTCTCATTCCTGCTTGTG ACTTGGTGGTTTGCTACGAC
**TGF-β**	193	Forward Reverse	AATTCCTGGCGTTACCTTGG GGCTGATCCCGTTGATTTCC
**IFN-γ**	201	Forward Reverse	AGGAACTGGCAAAAGGATGG GACCTCAAACTTGGCAATACTC
**GAPDH**	224	Forward Reverse	CGGTGTGAACGGATTTGG CTCGCTCCTGGAAGATGG

Bax: Bcl-2-associated X protein, BCL-2: B-cell lymphoma 2, TNF-α: Tumor Necrosis Factor-alpha, TGF-β: Transforming growth factor-beta, IFN-γ: Interferon-gamma, GAPDH; Glyceraldehyde-3-phosphate dehydrogenase

AFM: Atomic force microscopy

**Table 2:** Release of extract of ginger in different pH after 24 hr at 37 ^°^C

**pH**	**Release% (mean ± SEM)**
**5.5**	52.5 ±2.5
**6.5**	85.5 ±1.5
**7.4**	12.5 ±2.5

Gin: ginger extract, LipGin: liposomal ginger

Control: untreated, Dox: doxorubicin, Doxil: liposomal doxorubicin, Gin: ginger extract, LipGin: liposomal ginger

Control: untreated, Dox: doxorubicin, Doxil: liposomal doxorubicin, Gin: ginger extract, LipGin: liposomal ginger

Control: untreated, Dox: doxorubicin, Doxil: liposomal doxorubicin, Gin: ginger extract, LipGin: liposomal ginger

Control: untreated, Dox: doxorubicin, Doxil: liposomal doxorubicin, Gin: ginger extract, LipGin: liposomal ginger

Control: untreated, Dox: doxorubicin, Doxil: liposomal doxorubicin, Gin: ginger extract, LipGin: liposomal ginger

Control: untreated, Dox: doxorubicin, Doxil: liposomal doxorubicin, Gin: ginger extract, LipGin: liposomal ginger, TNF-α: tumor necrosis factor-α, TGF-β: transforming growth factor-β, IFN-γ: Interferon-γ

## Discussion

In this study, nanoliposomes containing ginger extract were prepared, and the anticancer and immune-stimulating effects on the growth of colorectal cancer cells were investigated *in vitro* and *in vivo*. The ginger extract nanoliposomes showed suitable mean particle size and PDI, good for targeted drug delivery to the tumor site through the EPR process (24). Also, measuring the zeta potential of the particles showed that they have a negative surface charge due to the presence of mPEG molecules on the surface of nanoliposomes, which improves blood circulation and extracellular matrix interaction stability of the drug delivery system in the bloodstream (25). Furthermore, ginger release from LipGin showed a significant difference between pH 6.5 (in tumor site) and pH 5.5 (in endosome) compared with pH 7.4 as physiological fluids pH, which proves effective targeted delivery of the extract to the tumor site (23). 

Mice treated with Gin and LipGin showed no weight loss during the treatment period. Also, liver and spleen functional markers, including BUN, Creatinine, SGOT, and SGPT, showed that nanoliposomes containing ginger extract were non-toxic to liver and kidney function. Similarly, a previous study in the mouse model of colorectal cancer showed insignificant BUN, creatinine, SGOT, and SGPT levels in the ginger extract-treated group compared with the control (9). Ginger extract has protective effects against induced hepatotoxicity and nephrotoxicity by various toxicants (26, 27). In this respect, the effect of ginger on carbon tetrachloride-induced hepatotoxicity (CCl4) in rats proved it’s effective in improving hepato-renal toxicity. Although Dox has cardiotoxicity, it has been used as a positive control with a defined concentration based on previous articles. So, its toxicity has not been tested again in this study (28).

The results of MTT showed the non-toxic effect of nanoliposomes containing ginger extract on mouse splenocytes as a normal cell, suggesting its quite low adverse effects on normal cells. Also, MTT on the C26 cell line showed that LipGin was less toxic than Gin in the mouse colorectal cancer cell line, which could be due to the slower release of ginger extract from nanoliposomes at pH 7.4. A study in 2012 showed that the liposomal form of thymoquinone was less toxic than its free form on MCF7 and T47D breast cancer cells, which was consistent with our study (29). IC50 values for LipGin and Gin groups were 240 and 170 μg/ml, respectively. The lower IC50 value of Gin probably is due to faster dissolution and higher *in vitro* cytotoxicity of ginger molecules. 

H&E staining of the tumor tissue sections showed an increase in TILs toward the tumor site, more significant in Gin and LipGin groups. Also, an increase in TILs number was more significant in the LipGin group compared with the Gin group. Flow cytometry results showed a considerable increase in CTLs compared with the control in tumor tissue. According to the results of lymphocyte count in tumor tissue sections, it can be concluded that the increase in the lymphocyte number in the tumor tissue was due to the increase in T-cytotoxic lymphocytes.

This study showed a significant increase in the expression of the pro-apoptotic Bax gene in all groups. Also, Bax gene expression was more significant in the LipGin than in the Gin group. Bcl-2 gene expression as an anti-apoptotic molecule decreased in all treated groups; however, it was significant in doxorubicin and Doxil groups. Considering the regulatory role of Bax and Bcl-2 genes in the process of apoptosis, these results indicated the increasing anti-proliferative effect of LipGin compared with Gin in a mouse model of colorectal cancer. A study in 2011 showed that the liposomal oxaliplatin increased the Bax gene expression compared with non-liposomal oxaliplatin in the mouse model, which is similar to the present study results (30). 

One of the aims of this study was to evaluate the immune-stimulating efficacy of the prepared nanoliposomes. TNF-α gene expression showed insignificant differences between different groups. However, TGF-β gene expression decreased significantly in all groups compared with the control. A study in 2021 showed that gingerol, as one of the active components of ginger, reduces the expression of the TGF-β gene in mice with breast cancer (31). The multifunctional cytokine TGF-β has been shown to have several pro-tumorigenic functions during cancer progression (32). TGF-β suppresses the adaptive immune response during cancer progression by inhibiting T cells’ activation, proliferation, differentiation, and migration (33). Also, up-regulation of TGF-β expression was correlated with metastasis and poor prognosis in patients with colorectal cancer (34). IFN-γ gene expression increased significantly in Doxil, Gin, and LipGin groups compared with the control. Also, IFN-γ gene expression was more significant in the LipGin group compared with the Gin group. A study in 2015 showed that deficiency of IFN-γ or its receptor promotes colorectal cancer development (35). IFN-γ, a cytokine secreted by activated T cells and natural killer cells, has anticancer effects, including enhancing MHC-II antigen processing and presentation (36). These results showed nanoliposomes containing ginger extract exert their antitumor effects by altering the cytokine pattern, including IFN-γ and TGF-β, in the early stages of colon cancer.

This study showed significantly reduced tumor size of mice in Gin and LipGin groups compared with other groups. Nanoliposomes containing ginger extract reduced tumor size compared with ginger extract; however, it was insignificant. Similarly, a study comparing the liposomal and non-liposomal forms of Pemetrexed in a mouse model of breast cancer showed no difference in tumor size (37). Therefore, due to the stronger antitumor response of the liposomal form of ginger than ginger at the molecular and cellular levels including cytotoxic T lymphocytes in tumor tissue and IFN-γ and Bax molecules, the reason for the lack of significant differences between the two groups in tumor size can be evaluated by increasing the treatment duration in future studies.

 In addition, the tumor weight decreased significantly in Doxil and LipGin groups compared with the control; however, there was no significant difference in tumor weight between Gin and LipGin, and more research is needed to understand the other cellular mechanisms responsible for the anticancer effects of liposomal ginger.

## Conclusion

In this study, the extracted ginger was successfully encapsulated in nanoliposomes. Zeta potential and particle size of the nanoliposomes indicated the suitability of this formulation for targeted drug delivery to the tumor site through the EPR process. Also, the higher release percentage of ginger extract in PH 6.5 as the pH of tumor tissue indicated effective and targeted delivery of the extract to the tumor site. Furthermore, our data demonstrated that liposomes containing ginger extract enhanced anticancer properties by increasing the induction of apoptosis and stimulating the immune system. Therefore, the proposed formulation might be considered a promising solution for active targeted drug delivery and can be used in future clinical trials.

## Limitations of the study

An *in vivo* study to evaluate the biological distribution of liposomes is necessary.

## Authors’ Contributions

AG provided concept, methodology, and software analysis; MY helped with data curation and original draft preparation; MRJ and FM helped visualize and investigate; GM provided software analysis and validation; AGh helped write, review, and edit.

## Conflicts of Interest

The authors report no conflicts of interest. 
